# A Fenton-like cation can improve arsenic trioxide treatment of sclerodermatous chronic Graft-versus-Host Disease in mice

**DOI:** 10.3389/fimmu.2022.917739

**Published:** 2022-08-09

**Authors:** Charlotte Chêne, Mohamed Maxime Jeljeli, Dominique Rongvaux-Gaïda, Marine Thomas, François Rieger, Frédéric Batteux, Carole Nicco

**Affiliations:** ^1^ Département 3I Infection, Immunité et Inflammation , Institut Cochin, INSERM U1016, Université de Paris, Paris, France; ^2^ MEDSENIC SAS, Strasbourg, France; ^3^ Université de Paris, Faculté de Médecine, AP-HP-Centre Université de Paris, Hôpital Cochin, Service d’immunologie biologique, Paris, France

**Keywords:** arsenic, copper, reactive oxygen species, fibrosis, chronic GvHD

## Abstract

Graft-versus Host Disease (GvHD) is a major complication of hematopoietic stem cell transplant. GvHD is characterized by the chronic activation of immune cells leading to the development of systemic inflammation, autoimmunity, fibrosis and eventually death. Arsenic trioxide (ATO) is a therapeutic agent under clinical trial for the treatment of patients with systemic lupus erythematosus (SLE) and chronic GvHD (cGvHD). This therapy is admittedly rather safe although adverse effects can occur and may necessitate short interruptions of the treatment. The aim of this study was to combine ATO with a divalent cation, to generate a Fenton or Fenton-like reaction in order to potentiate the deletion of activated immune cells through the reactive oxygen species (ROS)-mediated effects of ATO in a mouse model, and thereby enabling the use of lower and safer ATO concentrations to treat patients with cGvHD. *In vitro*, among the various combinations of divalent cations tested, we observed that the combination of ATO and CuCl_2_ (copper chloride) induced a high level of oxidative stress in HL-60 and A20 cells. In addition, this co-treatment also decreased the proliferation of CD4^+^ T lymphocytes during a mixed lymphocyte reaction (MLR). *In vivo*, in a cGvHD mouse model, daily injections of ATO 2.5 µg/g + CuCl_2_ 0.5 µg/g induce a decrease in lymphocyte activation and fibrosis that was equivalent to that induced by ATO 5 µg/g. Our results show that the addition of CuCl_2_ improved the effects of ATO and significantly limited the development of the disease. This co-treatment could be a real benefit in human patients to substantially decrease the known ATO side effects and optimize ATO treatment in pathologies characterized by activated cells sensitive to an increase in oxidative stress.

## Graphical Abstract

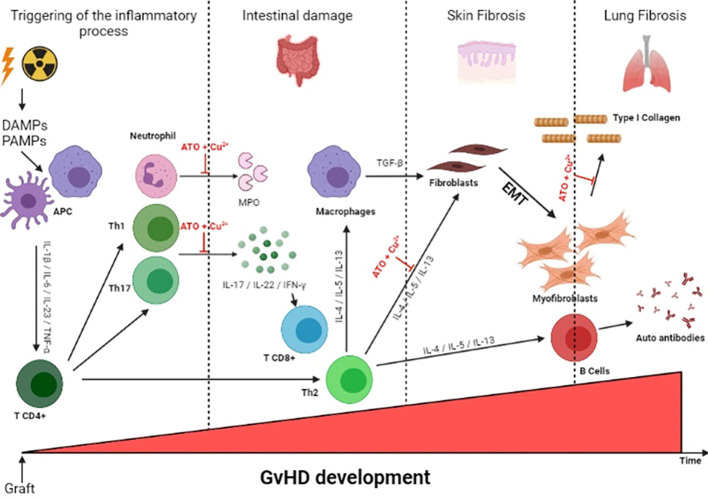

## Introduction

Allogeneic hematopoietic stem cell transplantation (allo-HSCT) is used to treat many malignant and non-malignant hematological disorders (1, 2). The recipient’s immune system is usually destroyed with radiation or chemotherapy before the transplantation. GvHD occurs when immunocompetent donor T cells recognize the recipient host as foreign and mount an immune response to allogeneic antigen-bearing cells, with subsequent destruction of host tissues. GvHD, whether acute or chronic, occurs in 20-50% of cases (3) and remains the most common complication after transplantation in patients (4). Acute GvHD primarily affects epithelial tissue and benefits from a number of therapeutic initiatives, whereas chronic GvHD (cGvHD) is a systemic disease which remains difficult to treat. cGvHD may involve a single organ or several tracts, such as skin, mouth, gastrointestinal tract, liver, lungs, musculoskeletal tract, joint and fascia, eyes and lymphohematopoietic and genital tracts (5, 6). Although the pathogenesis of cGvHD remains poorly understood, numerous studies have highlighted the role of donor immune cells (7, 8). cGvHD is the result of an uncontrolled alloreactive reaction between cells of the donor immune system and those of the recipient (9, 10). Corticosteroids are the current prophylactic strategies of care but only about 40% of patients have a durable response and corticosteroids can induce severe complications (11). Experimental models have greatly improved our understanding of the pathogenesis of GvHD and have led to newer approaches targeting different components of immune dysregulation. ATO is a drug recognized in the treatment of acute promyelocytic leukemia (12, 13) and is also a therapeutic alternative tested in clinical studies in acute (14, 15) and chronic GvHD (16, 17) but also lupus progression (18). In a mouse model of cGvHD, the administration of ATO increased the production of reactive oxygen species (ROS), especially H_2_O_2_ and hydroxyl radicals (16), which induced apoptosis of activated alloreactive lymphocytes and plasmacytoid dendritic cells (19), leading to a reduction of systemic inflammation and fibrosis of the skin and lung (16). The Fenton reaction is used as a source of hydroxyl radicals and is an initiator of biological damage (20, 21). The term ‘Fenton-like’ is used to describe comparable reactions due to other transition metals, such as copper (22), that are able to induce ROS production. Fenton and Fenton-like reaction-based chemodynamic therapy (23) consists of new strategies to enhance anticancer drug efficacy due to their capacity to generate an oxidative burst that activates cell death. In the current study, we evaluated the effect of a Fenton-like reaction induced by divalent cations combined with ATO treatment with the aim of increasing ROS production to favor the selective deletion of activated lymphocytes as they represent key elements in the GvHD pathophysiological process. Associating divalent cations with ATO treatment and enhancing the biological effects of ATO could help to reduce its dosage and potential side effects. To that end, we evaluated the association of various divalent cation salts with ATO. Copper was selected *in vitro* because of its synergistic effect on ROS-induced cell death when associated with ATO. *In vivo*, this combination was found to be efficient in a B10.D2 to BALB/c cGvHD mouse model.

## Materials and methods

### Chemicals

The first five divalent cations tested were purchased from Sigma-Aldrich (Saint-Quentin Fallavier, France): CuSO_4_, FeSO_4_, MnSO_4_, ZnSO_4_ and AuCl_2_. The three other divalent cations tested were obtained from ChemCon (Freiburg, Germany): CuCl_2_, MnCl_2_ and ZnCl_2_ (GMP grade). Arsenic trioxide (Arscimed, clinical grade batch, stock solution at 1 mg/ml) was obtained from MEDSENIC (Strasbourg, France).

### Cell lines

HL-60, a human Caucasian promyelocytic leukemia cell line, was purchased from ATCC (n°CCL-240). Cells were maintained throughout the experiments in a 75-cm^2^ flask (Falcon 250 mL 75 cm^2^, #353135) in RPMI 1640 + GlutaMax culture medium (Sigma-Aldrich, Saint-Quentin- Fallavier, France) containing 10% fetal bovine serum (FBS) (Gibco, USA), 1% penicillin-streptomycin (Gibco, USA), 1% ciprofloxacine (Fresenius Kabi, France) and 1% fungizone (Gibco, USA).

A20 is a mouse cell line (ATCC TIB-208). It was cultured in a 75-cm^2^ flask (Falcon 250 mL 75 cm^2^, #353135) in RPMI 1640 + GlutaMax culture medium (Sigma-Aldrich, Saint-Quentin- Fallavier, France) containing 10% FBS (Gibco, USA), 1% penicillin-streptomycin (Gibco, USA), 1% ciprofloxacine (Fresenius Kabi, France), 1% amphotericin B (Gibco, USA) and 1% 2-mercaptoethanol (Gibco, USA).

### Cell cultures

Cells were seeded in 96-well flat-bottom plates (Falcon, Corning, #353077) and incubated for 48 hours under different experimental conditions at 37°C and 5% CO_2_. Two plates were prepared in parallel to measure H_2_O_2_ production, glutathione (GSH) production and cell viability.

### Measurement of H_2_O_2_ production

After 48 hours of treatment with ATO ± Fenton-like molecule, the supernatant was removed and 50 µL per well of 50 µg/mL 2’, 7’-dichlorodihydrofluorescein di-acetate (Sigma-Aldrich, Saint-Quentin- Fallavier, France) in PBS was added.

The H_2_O_2_ production was assessed by spectrofluorimetry using a Fusion spectrofluorometer (Packard). Fluorescence intensity was recorded immediately (T0 hours) and after 6 hours of incubation (T6 hours). Fluorescence excitation/emission maxima were for 2’, 7’-dichlorodihydrofluorescein diacetate at wavelengths of 485 and 530 nm in arbitrary units (AU).

### Measurement of GSH production

After 48 hours of treatment with ATO ± Fenton-like molecule, the supernatant was removed and 50 µL per well of 50 µg/mL monochlorobimane (Sigma-Aldrich, Saint-Quentin- Fallavier, France) in PBS was added.

The GSH production was assessed by spectrofluorimetry using a Fusion spectrofluorometer (Packard). Fluorescence intensity was recorded immediately (T0 hours) and after 6 hours of incubation (T6 hours). Fluorescence excitation/emission maxima were, for monochlorobimane, 380/461 nm.

### Cell viability

The medium was removed and cells were stained with 0.5% crystal violet and 30% ethanol in PBS for 30 minutes at room temperature. After two PBS washes, methanol was added to the cell pellet, and absorbance was measured at 550 nm using a Fusion spectrofluorometer (24).

### Mixed lymphocyte reaction

The model is based on the cultured suspensions of spleen cells from a female C57Bl/6 mouse as responder cells and from a female BALB/c mouse as stimulator cells. The stimulator cells were irradiated at 30 Gy. The spleen cells were mechanically separated and the erythrocytes were eliminated by hypotonic lysis with ammonium chloride potassium (ACK – NH_4_Cl 0.15 M + KHCO_3_ 1 mM + Na_2_EDTA 0.1 mM). The assay included appropriate negative controls (C57Bl/6 splenocytes and irradiated BALB/c splenocytes seeded alone) and positive control cultures (C57Bl/6 splenocytes stimulated with 5 µg/ml of anti-CD3 (Ref 14-0032-86, eBioscience) and 2 µg/ml anti-CD28 (Ref 14-0281-86, eBioscience).

### Cell proliferation measurement

The UptiBlue Viable Cell Counting assay was used to quantify the *in vitro* cell proliferation. C57Bl/6 cells (responding cells) were seeded with irradiated BALB/c cells (stimulating cells) at 6x10^5^ per well per line in 96-well round-bottom plates (Falcon, Corning, reference: 353077). Cells were cultured on complete medium containing RPMI 1640 + 10% FBS and antibiotics (**Figure 2A**). The mixed cell culture was incubated for 48 hours under various treatments in an incubator at 37°C and 5% CO_2_. *N*-acetylcysteine (NAC) was added at different concentrations in the culture medium to inhibit the pro-oxidant effect of ATO and copper.

After 48 h of treatment, 10 µl of UptiBlue Viable Cell Counting assay (Interchim, reference: UP669413) were added directly to the culture medium. C57Bl/6 cell proliferation was measured 24 hours afterwards, using a Fusion spectrofluorimeter (microplate reader fluorometer, Packard).

### Animals

Eight-week-old female BALB/c mice (H2^d^) were purchased from Janvier Laboratory (Le Genest-Saint-Isle, France) and male B10.D2 mice (H2^d^) were kindly offered by Colette Kanellopoulos-Langevin, CDTA-CNRS-Orléans, France). Mice were maintained with food and water ad libitum. The animals were given humane care, according to the guidelines of our institution (Université Paris Descartes, Paris). The protocols and all experimental procedures of this study were approved by the Ethics Committee of Paris Descartes University (Animal facilities C75-14-05, DAP 16-026 # 8233).

### Experimental procedure for cGvHD induction

Recipient mice were irradiated with 7.50 Gy from a Gammacel [137Cs] source. After three hours, they were injected in the retro-orbital vein with B10.D2 spleen cells (2.10^6^ per recipient) and bone marrow cells (10^6^ per recipient) as previously described (16). A control group of BALB/c recipient mice received syngeneic cells from other BALB/c donor mice (syngeneic spleen cells and bone marrow cells). An irradiation control group was irradiated without receiving any graft of cells. These mice were irradiation controls and were expected to die after 10-12 days. The evolution of the disease followed the standard curve (25); the mice were therefore sacrificed at the usual endpoint of 42 days. Indeed, it is at this time that the symptoms of GvHD are optimal for the study while remaining tolerable for the animal.

### Subcutaneous injection of A20 cells in BALB/c AnN mice

A20 cells were resuspended at a concentration of 2.10^6^ cells in 100 µL of PBS and injected into the shaved back of mice. Mice were divided into five groups of n=9 mice per group. Animals were monitored daily for survival and twice a week with a microcaliper to measure tumor size, calculated as (length) x (width)^2^ x 0.5, as previously described (26). Mice showing signs of distress or suffering from the tumor were humanely euthanized. Mice were sacrificed at day 32.

### Treatment of cGvHD mice and BALB/c AnN mice

cGvHD mice and BALB/c AnN mice were divided into five groups. One group (cGvHD mice or A20-PBS) received PBS as a treatment and the four remaining groups received a daily injection of ATO alone 2.5 µg/g or 5 µg/g, ATO 2.5 µg/g with CuCl_2_ 0.5 µg/g, or CuCl_2_ alone 0.5 µg/g.

### Clinical signs

Alopecia score: To determine the alopecia score, we assigned a score to each mouse using the following criteria: 0: no alopecia; 0.5: piloerection on back; 1: alopecia <1 cm^2^; 2: alopecia >1 cm^2^. Two scientists blinded to the experimental group assignment recorded the incidence and severity score every week. The results shown correspond to the results on day 42.

Weight: Mice were weighed every other day throughout the experiment to verify that the treatments were not toxic. These histogram results correspond to day 42 measurements (**Figure 4D**).

ALT (alanine aminotransferase): Serum level of ALT was used as a marker of hepatocyte cytolysis and was quantified using a standard automated clinical chemistry analyzer (Modular PP, Roche Diagnostics, Meylan, France). These histogram results correspond to day 42 measurements (**Figure 4E**).

### Assessment of fibrosis

Skin thickness: Mice skin thickness was measured every week with a calliper and one day before sacrifice. These histogram results correspond to day 41 measurements (**Figure 5C**).

Histopathological analysis: skin, lung and liver pieces fixed in formol were embedded in paraffin. Tissue sections 5 µm thick were prepared and then stained with H&E (hematoxylin and eosin) or Sirius red. The slides were examined by standard bright field microscopy (Nikon eclipse 80i).

### Isolation and stimulation of spleen cells

Mice were sacrificed by cervical dislocation. Cellular splenic suspensions were prepared after hypotonic lysis of erythrocytes in potassium acetate solution and three washes in complete RPMI medium completed with 10% heat inactivated FBS, 1% streptomycin-penicillin, 1% sodium pyruvate, 1% ciprofloxacin and 1% amphotericin B.

### Reverse transcription - quantitative PCR (RT-qPCR)

Total RNA was isolated using Trizol reagent (Ref 15596018, Ambion), according to the manufacturer’s protocol. The expression levels of *collagen I*, *α-SMA*, *IFN-γ*, *MPO*, *IL-13*, *CD45*, *GAPDH* and *β-actin* were evaluated by RT-qPCR in different organs. A QuantiTect SYBR^®^ Green RT-qPCR Kit (Ref 04053228014782, Qiagen) (**Figure 7C**) on a LightCycler 480 II instrument (Roche Applied Science, France) was used to perform one-step RT-qPCR. Samples were normalized to mRNA expression of housekeeping genes (*β-actin* for intestine and lung and *GAPDH* for skin), and results were expressed as fold increase using the formula 2^−˄˄Ct^. Primers used for PCR are listed in **Supplementary Table 1**.

### Flow cytometry analysis

Splenocytes were prepared as described above. Cells were then incubated with the appropriate antibody at 4°C for 30 min in the dark in PBS with 2% FBS. Flow cytometry was performed using a FACS Fortessa II flow cytometer (BD Biosciences), according to standard techniques. For the characterization of splenic cells, the monoclonal antibodies used were: CD3-AF700 (17A2, #100216), CD4-BV510 (GK1.5, #100449), CD8-AF647 (53-6.7, #100727), CD25-PE conjugated (PC61.5, #12-0251-81), B220-BV421 (RA3-6B2, #562922), CD62l-PeCy5 (MEL-14, #104410), CD44-BV605 (IM7, #103047), CD11b-PerCPcy5.5 (M1/70, #101228), CD80-PE (16-10A1, #09605B), F4/80-BV711 (BM8, #123147), CD86-FITC (GL-1, #105005), CD206-AF647 (MR5D3, #565250), CD43-BV421 (S7, #562958), CD11c-AF700 (N418, #117320), MHCII-EFluor480 (M5/114.15.2, #48-5321-82) from eBiosciences (Thermo Fisher Scientific, Villebon-Sur-Yvette, France). Data were analyzed with FlowJo software (Tree Star, Ashland, OR).

### Statistical analysis

The results were analyzed with the GraphPad Prism8 program. The one-way test ANOVA with Bonferroni’s correction was used to determine the differences between two experimental groups. A difference with P< 0.05 was considered as significant.

## Results

### Effects of ATO on ROS production and on cell viability of HL-60 cells

**Figure 1:**
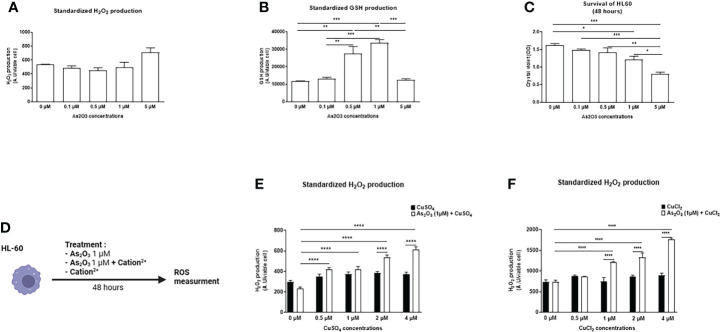
Effects of ATO ± Cu^2+^ on H_2_O_2_ and GSH levels and cell viability on HL-60 cell line. **(A)** Standardized H_2_O_2_ level produced by HL-60 cells in culture after treatment during 48 hours with increasing concentrations of ATO (0 to 5 µM). H_2_DCFDA was measured by spectrofluorometry and the results (AU) were divided by cell viability established by colorimetry (crystal violet). **(B)** Standardized GSH level produced by HL-60 cells in culture after treatment during 48 hours with increasing concentrations of ATO (0 to 5 µM). The results obtained by spectrofluorometry using monochlorobimane were divided by cell viability established by colorimetry (crystal violet). **(C)** Cell viability of HL-60 cells in culture measured after treatment during 48 hours with increasing concentrations of ATO (absorbance at 550 nm) using a Fusion spectrofluorometer. **(D)** Schematic representation of the *in vitro* experiment on HL-60 cells. The cells were treated for 48 hours with ATO at 1 μM +/- different divalent cations. The production of hydrogen peroxide was measured by spectrofluorometry. **(E, F)** Standardized H_2_O_2_ level produced by HL-60 cells in culture after treatment during 48 hours with one concentration of ATO (1 µM) and with increasing concentrations of CuSO_4_/CuCl_2_ (0-4 µM). H_2_DCFDA was measured by spectrofluorometry and the results (AU) were divided par cell viability established by colorimetry (crystal violet). NS: not significant; *p <0.05; **p<0.01; ***p<0.001; ****p<0.0001. The results are the mean of 6 replicates per sample.

Different doses of ATO were tested on these cells in order to measure the production of ROS and to observe the toxicity of ATO to define an optimal dose for the rest of the experiments. H_2_O_2_ production did not vary regardless of the ATO concentration. However, ATO at a high concentration (5 μM) significantly decreased GSH production and cell viability (p<0.0001). Conversely, at 1 µM, ATO significantly increased GSH production (p<0.0001) and decreased by 25% cell survival of HL-60 cells (p=0.0144) (**Figures 1A–C**). Collectively, these results allowed us to choose a low dose of ATO that could be potentiated by a divalent cation and thus limit the undesirable effects of high doses of ATO.

### Addition of a divalent cation to ATO changed the oxidation state of HL-60 and A20 cells

**Figure 2:**
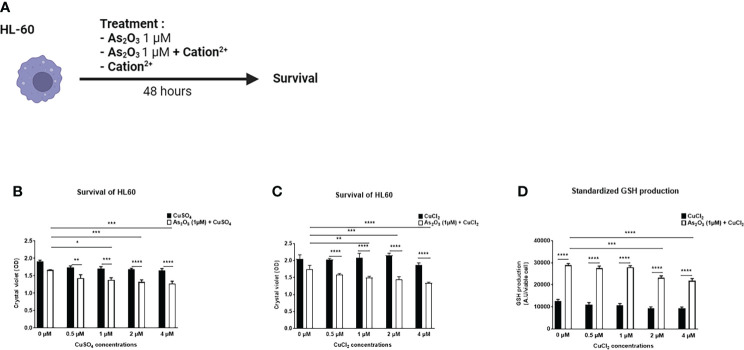
Effect of ATO ± Cu^2+^ on cell survival and on GSH production of HL-60 cell line. **(A)** Schematic representation of the *in vitro* experiment on HL-60 cells. The cells were treated for 48 hours with arsenic trioxide at 1 μM +/- different divalent cations. The survival of HL-60 cells was measured by absorbance. **(B, C)** Cell viability of HL-60 cells in culture measured after treatment during 48 hours with one concentration of ATO (1µM) and with increasing concentrations of CuSO_4_/CuCl_2_ (0-4 µM). **(D)** Standardized GSH level produced by HL-60 cells in culture after treatment during 48 hours with one concentration of ATO (1 µM) and with increasing concentrations of CuCl_2_ (0-4 µM) The results obtained by spectrofluorometry using monochlorobimane were divided by cell viability established by colorimetry (crystal violet). NS: not significant; *p<0.05; **p<0.01; ***p<0.001; ****p<0.0001. The results are the mean of 6 replicates per sample.

We evaluated the effects of several divalent cations (CuSO_4_, FeSO_4_, MnSO_4_, ZnSO_4_, AuCl_2_, CuCl_2_, MnCl_2_, ZnCl_2_), able to potentiate the low dose of ATO (1 µM), on the production of H_2_O_2_ by HL-60 and A20 cells (**Figure 1D**). CuSO_4_ and CuCl_2_ increased H_2_O_2_ production when added to ATO. This effect was dose dependent and adding copper to ATO significantly increased H_2_O_2_ production compared to 1 µM ATO alone (p<0.0001). We also showed that this effect was increased, compared to CuSO_4_ and CuCl_2_ alone, by 39% (p<0.0001) when we added 2 µM of CuSO_4_ and by 62% (p<0.0001) when we added 1 µM of CuCl_2_ to ATO (**Figures 1E, F**). The effects of the other cations are shown in the **Supplementary Material**. Briefly, FeSO_4_ and AuCl_2_ increased H_2_O_2_ production when used at a high concentration (4 µM, p<0.0001 and 1 µM, p<0.001, respectively) but this effect was not significantly greater than with FeSO_4_ or AuCl_2_ alone. Addition of manganese to ATO only increased H_2_O_2_ production at the highest concentration of 1 µM by 44% for MnSO_4_ and by 58% for MnCl_2_ (p<0.0001). ZnSO_4_ potentiated the effect of ATO when added to concentrations from 12.5 µM to 50 µM of ZnSO_4_ (p=0.0208, p=0.0015 and p<0.0001, respectively). On the contrary, ZnCl_2_ had no effect on H_2_O_2_ production (**Supplementary Figures 1A–F**). Concerning the effect of our divalent cations associated with ATO 1 µM on the survival of HL-60 cells (**Figure 2A**), only CuSO_4_ and CuCl_2_ had an effect at any of the tested concentrations. CuSO_4_ + ATO significantly decreased the viability of HL-60 at 1 µM (by 19%, p=0.0003), 2 µM (by 21%, p<0.0001) and 4 µM (by 23%, p<0.0001) compared to copper alone and accentuated the effect of ATO alone (p=0.0194, p=0.0009 and p=0.0001, respectively). Adding CuCl_2_ to ATO decreased the viability at the same concentrations: 1 µM (by 28%, p<0.0001), 2 µM (by 33%, p<0.0001) and 4 µM (by 29%, p<0.0001) compared to CuCl_2_ alone (**Figures 2B, C**, respectively). All survival results for other cations are shown in the Supplementary Material (**Supplementary Figures 2A–F**). Following these results, we opted for copper as a divalent cation to potentiate the effect of ATO in our *in vivo* experiments. We chose CuCl_2_ over CuSO_4_ because its effect on the production of H_2_O_2_ was higher (mean: 612.211 AU vs 1771.61 AU).

We then evaluated the effect of CuCl_2_ and ATO on the antioxidant GSH. We observed that when 2 or 4 µM CuCl_2_ were added to 1 µM ATO, GSH production was significantly decreased compared to ATO alone (mean: 23123.8 AU, p=0.0001 and mean: 21697.9 AU, p<0.0001, respectively) (**Figure 2D**).

Similarly, as for HL-60 cells, the co-treatment increased A20 cell production of H_2_O_2_ (p<0.0001) (**Supplementary Figure 3A**). We observed a decrease in GSH production when adding CuCl_2_, starting from 1 µM (p<0.0001) (**Supplementary Figure 3B**). A decrease in cell survival was also observed when the cells were treated with ATO alone at 1 μM or with ATO and CuCl_2_ at different doses. However, Cu^2+^ did not change the decrease in cell survival compared to ATO alone (**Supplementary Figure 3C**).

### ATO and Cu^2+^ decreased lymphocyte proliferation during a mixed lymphocyte reaction

**Figure 3:**
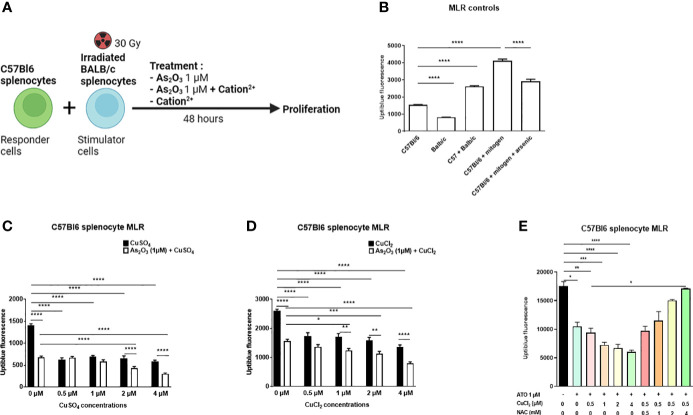
ATO and Cu^2+^ decrease lymphocyte proliferation during Mixed Lymphocytes Reaction (MLR). **(A)** Schematic representation of the MLR. Splenocytes from C57B1/6 mice were co-cultured with previously irradiated splenocytes from BALB/c mice. The co-culture was treated for 48 hours with arsenic trioxide at 1 μM +/- the Cu^2+^. After stimulation, the proliferation of the C57Bl/6 cells was measured using the UptiBlue test. **(B)** UptiBlue fluorescence analysis of lymphocyte cell proliferation after *in vitro* MLR. Responder cells consisted of lymphocytes derived from C57Bl/6 mice and stimulated cells consisted of irradiated lymphocytes from BALB/c mice. Cells were cultured in different conditions: C57Bl/6 cells alone, irradiated BALB/c cells alone, C57Bl/6 cells with BALB/c cells (MLR), C57Bl/6 cells with anti-CD3/CD8 antibody (mitogen) and C57Bl/6 cells with mitogen with ATO at 1 µM. **(C)** UptiBlue fluorescence analysis after MLR and treatment during 48 hours with one concentration of ATO (1 µM) and with increasing concentrations of CuSO_4_ (0-4 µM). **(D)** UptiBlue fluorescence analysis after MLR and treatment during 48 hours with one concentration of ATO (1µM) and with increasing concentrations of CuCl_2_ (0-4 µM). **(E)** UptiBlue fluorescence analysis after MLR and treatment during 48 hours with one concentration of ATO (1 µM), with increasing concentrations of CuCl_2_ (0-4 µM) and with or without increasing concentrations of NAC (0-4 Mm). *p <0.05; **p<0.01; ***p<0.001; ****p<0.0001. The results are the mean of 6 replicates per sample.

We performed a mixed lymphocyte reaction (MLR) mimicking *in vitro* the allo-immune response occurring during GvHD. The MLR allowed us to assess the effect of ATO and copper co-treatment on highly activated lymphocytes (**Figure 3A**). Splenocytes from C57Bl/6 mice were co-cultured only with splenocytes from BALB/c mice. No other stimulation was added to the co-culture. Addition of 1 μM ATO to the allogeneic reaction decreased the proliferation of the responding cells by approximately 29% compared to the positive control (anti-CD3/CD28) stimulation (p<0.0001) (**Figure 3B**). When Cu^2+^ was added at higher concentrations (2 μM and 4 μM), it potentiated the inhibition effect of ATO on C57Bl6 cell proliferation (p<0.0001 for CuSO_4_ and p=0.001 or p<0.0001 for CuCl_2_) (**Figures 3C, D**).

It has previously been reported that GvHD symptoms are abrogated in mice treated with ATO. This beneficial effect described by Kavian et al. involved oxidative stress balance in overactivated immune cells (16). Copper influenced the redox status of the cells *in vitro* through a Fenton-like reaction and optimized ATO treatment. We then evaluated the efficacy and safety of such a molecular association *in vivo* in a mouse model of sclerodermatous chronic GvHD. Briefly, irradiated BALB/c mice received bone marrow and spleen cells from B10.D2 mice. One week after the transplantation, the mice were treated, by intraperitoneal injections, with low-dose ATO (2.5 µg/g) or ATO (2.5 µg/g) + Cu^2+^ (0.5 µg/g) or high-dose ATO (5 µg/g) or Cu^2+^ (0.5 µg/g) alone. The development of the disease was evaluated until day 42, to include the inflammatory and the fibrotic periods of the pathology onset. Co-treatment with ATO + Cu^2+^ was expected to limit the development of the disease and, furthermore, allow the ATO dosage to be lowered and thereby reduce its potential adverse effects. In order to verify that the effect of ATO and copper on the MLR does indeed pass through oxidative stress, NAC was added to the culture medium. The addition of NAC inhibits the pro-oxidant effect induced by the co-treatment. A high concentration of NAC (4 mM) restored the proliferation of C57Bl/6 splenocytes (**Figure 3E**).

### *In vivo*, the association of Cu^2+^ with ATO decreases the clinical signs of cGvHD in mice

*In vitro*, we observed that the best condition to modulate the oxidative stress balance and cell proliferation was 1 µM of ATO with 4 µM of Cu^2+^. Therefore, the ratio 1 ATO to 4 Cu^2+^ was firstly retained to treat mice. Unfortunately, this dose of copper was hepatotoxic in BALB/c mice (data not shown). We then used copper concentrations comparable to dose used as a food supplement. BALB/c mice received injections of 0.2 μg/g and 0.5 μg/g 5 times a week for 5 weeks. At this dosage no hepatotoxic effects were observed, as assessed by monitoring alkaline phosphatase (PAL) and ALT serum levels in treated animals (data not shown). In the literature, the reported toxic side effects of ATO are nausea, vomiting, diarrhea, gastrointestinal hemorrhage, cerebral edema, tachycardia, dysrhythmias and hypovolemic shock (27). In addition, ATO may also affect reproduction and fetal development (28). Moreover, chronic exposure to arsenic is known to be a carcinogen in skin, bladder and lungs (29, 30). Therefore, the ability to lower the dosage of ATO to increase tolerance while maintaining its beneficial properties is of great interest.

**Figure 4:**
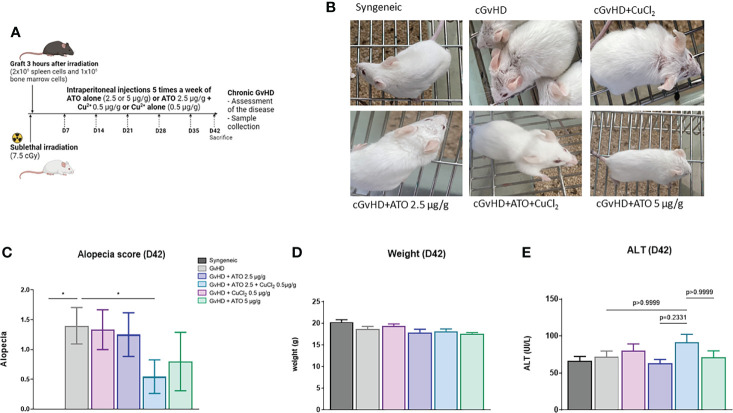
ATO and Cu^2+^ affect the clinical features of cGvHD in mice. **(A)** Schematic representation of the experimental induction of sclerodermatous cGvHD in mice. Receiver female (H2^d^) BALB/c mice were irradiated with 750 cGy and grafted intravenously with 2x10^6^ splenic cells and 1x10^6^ bone marrow cells from male (H2^d^) B10.D2 mice. Mice were monitored for survival, weight, and clinical signs of cGvHD for 42 days (day of sacrifice). **(B)** Photographs on day 42, showing alopecia during the development of cGvHD: mouse from the syngeneic group control, mouse from the cGvHD group control (PBS), mice from the cGvHD group treated by CuCl_2_ alone (0.5 µg/g), mice from the cGvHD group treated by low concentration of ATO (2.5 μg/g) + CuCl_2_ (0.5 μg/g) or ATO alone at 2 concentrations (2.5 and 5 μg/g). **(C)** The alopecia score was calculated based on the number of mice with alopecia noted by different grades and multiplied by the number of mice with alopecia at day 42. **(D)** Mice weight at day 42. **(E)** Levels of serum alanine aminotransferase (ALT) in the different experimental groups at day 42. *p<0.05. The results for each group are the mean of the measurements obtained per mouse: syngeneic (n = 7); cGvHD (n = 10); cGvHD-Cu (n = 6); cGvHD-ATO 2.5 µg/g (n = 8); cGvHD-ATO-Cu (n = 11); cGvHD-ATO 5 µg/g (n = 5).

The mean number of mice with alopecia in the cGvHD group was significantly higher than that in the control group (mean-syngeneic: 0 vs mean-GvHD: 1.4, p=0.03) (**Figure 4A**). Only the treatment with ATO+CuCl_2_ significantly reduced alopecia, by 61% (p=0.0368) (**Figures 4B, C**). The development of the disease and the different treatments did not induce any change in weight over time or liver damage (**Figures 4D, E** and **Supplementary Figure 4A**).

### ATO+Cu^2+^ works synergically to reduce dermal and visceral damage in cGvHD mice

#### Skin parameters

**Figure 5:**
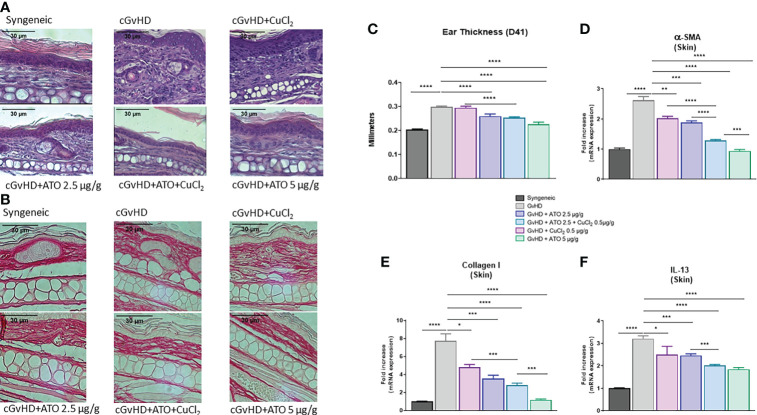
ATO and Cu^2+^ reduce skin fibrosis. **(A)** Hematoxylin and eosin (H&E) staining of ear skin sections (5 μm) (Eclipse 80i microscope; Nikon, original magnification ×20). Skin was disordered, with abundant infiltration of inflammatory cells and thickening (presentation of the 6 different groups of cGvHD mice). **(B)** Sirius red staining of ear skin sections (5 μm) (Eclipse 80i microscope; Nikon, original magnification ×20). Skin was filled with a collagen deposit. (Presentation of the 6 different groups of cGvHD mice). **(C)** Ear thickness of the different groups of cGvHD mice. The thickness of the ears was assessed weekly. Differences between groups were assessed at day 41, one day before sacrifice, to allow time for cGvHD to develop. **(D–F)** Relative mRNA expression of *α-SMA*, *collagen I* and *IL-13* in the skin of the ears. Data are presented as 2^(−ΔΔCT)^ relative to the levels of *GAPDH*. *p <0.05; **p<0.01; ***p<0.001; ****p<0.0001. The results for each group are the mean of the measurements obtained per mouse: syngeneic (n = 7); cGvHD (n = 10); cGvHD-Cu (n = 6); cGvHD-ATO 2.5 µg/g (n = 8); cGvHD-ATO-Cu (n = 11); cGvHD-ATO 5 µg/g (n = 5). *Ex vivo* measurements were realized in duplicate for each mouse.

Histologic examination showed that syngeneic mice did not present abnormal skin thickness or cell infiltration when compared to the cGvHD group. Treatment of mice with low and high doses of ATO (2.5 μg/g and 5 μg/g, respectively) limited skin thickening with a restoration of dermal layer organization. CuCl_2_ alone did not restore the dermal structure, whereas, after co-treatment with ATO+Cu^2+^, the skin presented less cellular infiltration (**Supplementary Figure 4B**) and dermal disorganization (**Figure 5A**). Staining of dermal biopsies with Sirius red evidenced that ATO treatment alone or associated with CuCl_2_ reduced the collagen deposit (**Figure 5B**). In the GvHD group, ear thickness was increased by 46% (p<0.0001) compared to the syngeneic group. ATO alone at 2.5 µg/g and 5 µg/g significantly reduced ear thickness, by 13% and 24% (p<0.0001 and p<0.0001, respectively). Adding copper to ATO decreased ear thickness by 15% compared to the untreated GvHD group (p<0.0001) (**Figure 5C**). Quantification of *α-SMA*, *collagen I* and *IL-13* gene expression, three markers of fibrosis, confirmed the clinical and histological observations. Indeed, CuCl_2_ associated with ATO decreased by 51% the expression of *α-SMA*, by 64% the expression of *collagen I* and by 37% the expression of *IL-13* in the skin (p<0.0001) but also potentiated the effect of ATO at a low dose (2.5 µg/g), by 32% and 18% for *α-SMA* (p<0.0001) and *IL-13* (p=0.0002), respectively (**Figures 5D–F**).

#### Lung parameters

**Figure 6:**
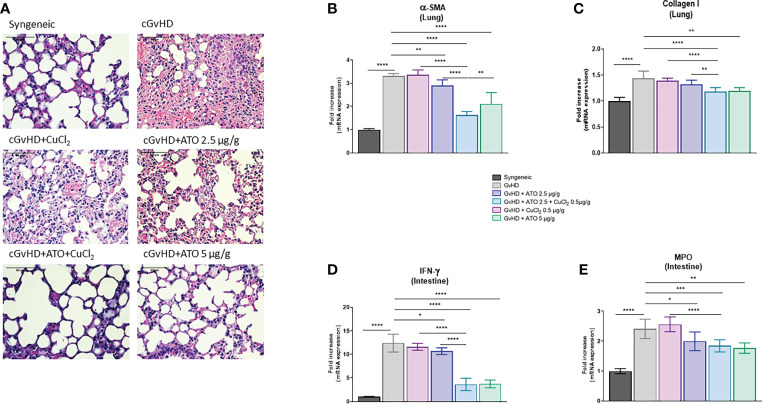
ATO and CuCl_2_ reduce lung fibrosis and intestine inflammation. **(A)** Hematoxylin and eosin (H&E) staining of lung sections (5 μm) (Eclipse 80i microscope; Nikon, original magnification ×20). Lung was disordered, with abundant infiltration of inflammatory cells and a reduction of intra-alveolar spaces (presentation of the 6 different groups of cGvHD mice). **(B, C)** Relative mRNA expression of *α-SMA* and *collagen I* in the lung. Data are presented as 2^(−ΔΔCT)^ relative to the levels of *β-actin*. **(D, E)** mRNA expression of *IFN-γ* and *MPO* in the intestine, relative to the levels of *β-actin*. Data are presented as 2^(−ΔΔCT)^. *p <0.05; **p<0.01; ***p<0.001; ****p<0.0001. The results for each group are the mean of the measurements obtained per mouse: syngeneic (n = 7); cGvHD (n = 10); cGvHD-Cu (n = 6); cGvHD-ATO 2.5 µg/g (n = 8); cGvHD-ATO-Cu (n = 11); cGvHD-ATO 5 µg/g (n = 5). *Ex vivo* measurements were realized in duplicate for each mouse.

We further assessed the effects of ATO and CuCl_2_ co-treatment on the pulmonary involvement of cGvHD. Histology of lung biopsies from cGvHD mice evidenced a decrease of intra-alveolar spaces along with an increase of cellular infiltration compared to the syngeneic group. Treatment with low-dose ATO (2.5 µg/g) alone or with CuCl_2_ alone did not restore the pulmonary alveoli nor did it reduce the infiltrate, whereas the high dose of ATO (5 µg/g) reversed the pulmonary damage (**Figure 6A**). The addition of CuCl_2_ to ATO was effective in alleviating the cGvHD lung lesions; thus, Cu^2+^ potentiated ATO at low doses. Fibrotic involvement of cGvHD was evidenced by pro-fibrotic markers’ mRNA expression. The CuCl_2_ and ATO association downregulated the expression of *α-SMA* by 51% (p<0.0001) and *Col1a1* by 18% (p<0.0001), compared to the untreated cGvHD group (**Figures 6B, C**, respectively). Adding copper to a low dose of ATO potentiated the effect of ATO alone (by 44%, p<0.0001 and by 11%, p=0.0015, respectively) or copper alone (by 52%, p<0.0001 and by 15%, p<0.0001, respectively).

#### Intestinal tract parameters

Finally, we analyzed markers that are overexpressed during gastrointestinal tract involvement in cGvHD. cGvHD mice presented a significantly increased mRNA expression of intestinal *IFN-γ* compared to the syngeneic group (mean-GvHD: 12.3977 AU vs mean-syngeneic: 1 AU) (p<0.0001). Treatment with low-dose ATO decreased the expression of *IFN-γ* by 14% (p=0.0382) (**Figure 6D**). Adding CuCl_2_ to ATO improved the beneficial effects of low-dose ATO alone and copper alone (p<0.0001), as it decreased *IFN-γ* production by 66% and 69%, respectively (**Figure 6D**). CuCl_2_ alone did not modulate the expression of the pro-inflammatory cytokine and thus did not by itself limit intestinal damage (**Figure 6D**). We observed an increase in *MPO* gene expression in the cGvHD control group compared to the syngeneic group (mean-GvHD: 2.4116 AU vs mean-syngeneic: 1 AU) (p<0.0001). Low-dose ATO reduced *MPO* expression by 17% (p=0.0288) compared to the control group (**Figure 6E**). Both ATO (5 µg/g) and ATO (2.5 µg/g) + CuCl_2_ (0.5 µg/g) moderated *MPO* expression, by 24% and 26%, respectively (p=0.0016 and p=0.0003, respectively) (**Figure 6E**).

### Effect of ATO+Cu^2+^ on immune status of mice

**Figure 7:**
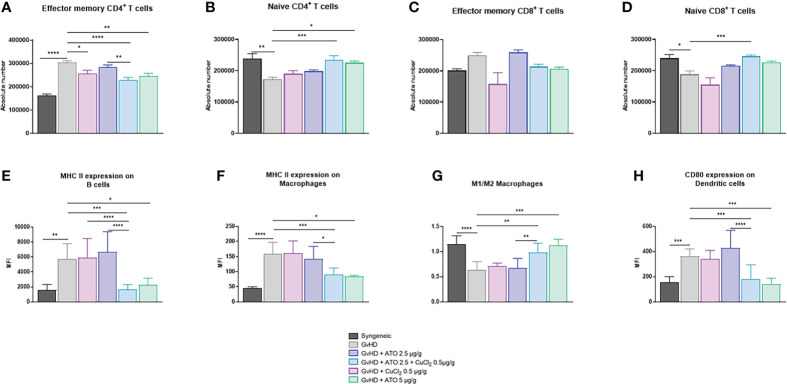
Modification of the adaptive and innate immune system by ATO and CuCl_2._ **(A–D)** Histograms showing the absolute count of spleen effector memory and naive CD4^+^ and CD8^+^ T cell populations. **(E)** Flow cytometry analysis of splenic B cells. **(FA–H)** Flow cytometry analysis of splenic macrophages and dendritic cells. Splenic T cells were gated on CD3^+^- and CD4^+^- or CD8^+^-positive cells. Splenic B cells were gated on CD3^-^ B220^+^. Macrophages were gated on F4/80^+^ CD11b^+^. Dendritic cells were gated on CD11c^+^ CD11b^+^. The gating strategy has been added as additional data (**Supplementary Figure 5**). *p <0.05; **p<0.01; ***p<0.001; ****p<0.0001. The results for each group are the mean of the measurements obtained per mouse: syngeneic (n = 7); cGvHD (n = 10); cGvHD-Cu (n = 6); cGvHD-ATO 2.5 µg/g (n = 8); cGvHD-ATO-Cu (n = 11); cGvHD-ATO 5 µg/g (n = 5).

The number of effector memory (CD44^+High^ CD62l^+Low^) CD4^+^ T lymphocytes was significantly increased in the cGvHD group compared to the control group (p<0.0001) and the numbers of naive (CD44^+Low^ CD62l^+High^) CD4^+^ T and CD8^+^ T lymphocytes were decreased (p<0.01 and p<0.05 respectively). High-dose ATO and the co-treatment ATO+CuCl_2_ markedly reduced the number of effector memory CD4^+^ T lymphocytes, by 19% and 25%, respectively (p<0.05, p<0.0001) and increased by 30% and 36% the number of naive CD4^+^ T lymphocytes, respectively (p<0.05 and p<0.001) (**Figures 7A, B**). The co-treatment also increased the number of naive CD4^+^ T lymphocytes by 31% (p<0.001) (**Figures 7C, D**). We observed an increase in the activation of B lymphocytes, represented by the expression of the surface marker MHC II in the untreated GvHD group compared to the syngeneic group (mean-syngeneic: 1578.67 Mean Fluorescence intensity (MFI); mean-GvHD: 5751.67 MFI) (p=0.0014). Only a treatment with a high dose of ATO or with ATO + CuCl_2_ significantly reduced abnormal B cell activation, by 60% and 71%, respectively (p=0.0457, p=0.0003, respectively) (**Figure 7E**). In addition, the number of activated MHC II^+^ macrophages increased in the cGvHD group compared to the healthy group (p<0.0001). Treatment limited the activation of macrophages with ATO 5 µg/g or co-treatment with copper by 47% and 33%, respectively (**Figure 7F**). We also observed a more pronounced balance in favor of increased M2 macrophages (Ly6c^+^), as reflected by the M1/M2 ratio in the cGvHD group compared to the syngeneic mice (mean-syngeneic: 1.14454 MFI; mean-GvHD: 0.6304 MFI) (p<0.0001). Treatment with high-dose ATO and co-treatment oriented macrophages to a clear M1 phenotype, as reflected by an increase of the M1/M2 ratio (mean-GvHD: 0.6304; mean-GvHD-ATO 5µg/g: 1.126; mean-GvHD-ATO+Cu^2+^: 0.985273 - p<0.05, p<0.001, respectively) (**Figure 7G**). The development of cGvHD also induced a marked activation of dendritic cells able to co-stimulate T lymphocytes through CD80^+^, unlike the syngeneic group (p<0.0001, p=0.0004). The high-dose ATO treatment as well as the co-treatment significantly reduced the expression of CD80^+^ cells (61% and 49%, respectively) (**Figure 7H**).

#### ATO+Cu^2+^ effect on B lymphoma in mice

ATO alone at high or low dose or in co-treatment with copper did not influence tumor development of B-cell lymphoma cells injected subcutaneously in all groups (**Supplementary Figures 6A, B**) (mean-A20: 200.375 mm^3^; mean-A20-ATO 5µg/g: 245.261 mm^3^; mean-A20-ATO+Cu^2+^: 151.067 mm^3^).

## Discussion

Arsenic trioxide (ATO) is a therapeutic agent used in the treatment of APL and under study to treat patients presenting chronic GvHD (NCT02966301/ClinicalTrial.gov and Phase 3 in preparation). An intravenous treatment with daily injections of ATO may provoke adverse, though reversible, effects on liver or cardiac physiology in humans, as also observed in a mouse model (31, 32). In the present study, we combined ATO with divalent cations to generate a Fenton-like reaction, in order to potentiate a low dosage of ATO treatment and make it usable for a longer period. We evidenced that ATO combined with Cu^2+^ can influence ROS production by cells and decrease allogeneic cell proliferation, *in vitro*, in an MLR. We also showed that, *in vivo*, Cu^2+^ can potentiate ATO effects on T cell activation and macrophage polarization in our murine model of sclerodermatous cGvHD, abrogating the clinical signs such as alopecia and decreasing the expression of early markers of fibrosis, thereby alleviating the visceral damage. Our co-treatment was well tolerated with no weight loss during the whole treatment period and no liver damage (**Figures 4D, E**).

We first showed a dose-dependent effect of ATO *in vitro* on human promyelocytic leukemia (PML) cell line (HL-60) viability and H_2_O_2_ and GSH production. It is well documented that ATO is an effective therapeutic cytotoxic agent able to induce apoptosis in PML (33). We selected 1 µM as a low dosage of ATO with a slight effect on the observed parameters in order to potentiate its effect with a divalent cation. We first evaluated the effects of several divalent cations (CuSO_4_, FeSO_4_, MnSO_4_, ZnSO_4_, AuCl_2_, CuCl_2_, MnCl_2_, ZnCl_2_), associated with ATO, on HL-60 and A20 tumor cells. We chose CuCl_2_ as the best Fenton-like divalent cation, because it potentiated the effects of ATO in optimizing H_2_O_2_ production, decreasing GSH activation and inducing HL-60 and A20 death at the same time, compared to the other tested cations. It is known that ATO by itself can induce apoptosis of a subtype of B-cell non-Hodgkin lymphoma (34). An improved effect of ATO was also reported on glioma cell lines when ATO was added concomitantly with ascorbic acid, in order to optimally modulate ROS production (35). Concerning Cu^2+^, Jembrek et al. evidenced its potent pro-oxidant capacity through the increase of ROS production and caspase 3/7 activity, which resulted in the reduction of tumor cell viability (36, 37). This specific effect of copper ions could explain the difference with other Fenton triggering ions, such as iron. In addition, a more efficient effect of copper on metal-catalyzed oxidation reactions has been already observed by Casciola-Rosen et al., and explained by a better diffusion of copper over iron within the cells (38). Moreover, it has been shown that this divalent cation could facilitate the passage of ATO into the cell through aquaporin channels, and in particular aquaporin-9, allowing it to act quickly and more efficiently (39). Therefore, in accordance with our results and the literature, we selected CuCl_2_ as the most promising divalent cation, among the ones we tested, to potentiate the pro-oxidant action of ATO. Chronic GvHD is the result of an uncontrolled alloreactive reaction of the donor immune system cells against the recipient (9, 10). We used an MLR in order to mimic, *in vitro*, the allo-immune response occurring during GvHD. We observed that the addition of Cu^2+^ potentiated the effect of ATO in reducing the proliferation of C57Bl/6 splenocytes. The addition of NAC to the culture medium significantly inhibited the effect of the co-treatment on the MLR. As we did not separate lymphocytes from other cell types, the decreased proliferation may not have been solely due to lymphocytes and might therefore be indirectly attributable to other cell types. However, MLRs are caused by direct activation of responder lymphocytes against MHC molecules on the recipient cells. In addition, our results are in agreement with previous studies that showed that ATO decreases the T cell response during MLR, as assessed by the production of IFN-γ and increased apoptosis of T cells in a dose-dependent manner (40, 41).

Our *in vitro* results allowed us to further assess the effect of the co-treatment of ATO and Cu^2+^ in a mouse model of chronic GvHD (42, 43). The co-treatment significantly reduced the GvHD phenotype of diseased mice. Alopecia and ear skin thickness, two of the main clinical signs of cGvHD, were significantly decreased in the ATO+Cu^2+^ group as in the ATO (2.5 µg/g) group. At a histological level, we observed a modification of the structure of the different skin layers, a thickening and an increase in skin collagen production (Sirius red collagen marker). These results are supported by the expression of the early markers of fibrosis, *α-SMA* and *IL-13*, which fell in the skin of co-treated mice. These results are fully in line with those obtained by Kavian et al. (16), who found that all these parameters were reduced by a high concentration ATO treatment. The association of copper with a low dose of ATO allowed us to obtain the same improvement as with a high dose for cutaneous fibrosis. Both lung histology and gene expression of *α-SMA* and *collagen I* evidenced that ATO+Cu^2+^ treatment can reduce pulmonary fibrosis with preservation of the pulmonary alveoli, and a decrease in cellular infiltrate and the accumulation of collagen. These results are in agreement with the work of Luo et al., who evidenced that ATO can reduce the fibrosis caused by injections of bleomycin, in particular by decreasing the expression of *α-SMA* and *collagen I* in lungs (44). Decreasing the dose of ATO and adding a low dose of copper preserves the effect of a high dose of ATO in the development of the disease, as reflected by less exacerbated fibrosis of the lungs. In addition to limiting cutaneous and pulmonary fibrosis, ATO+Cu^2+^ treatment decreased the intestinal damage induced by cGvHD. We showed that the association of Cu^2+^ with ATO can further reduce the expression of *IFN-γ*, which is known to be involved in the intestinal damage observed in GvHD (45, 46). In a mouse model of inflammatory bowel disease (IBD), it has been shown that *myeloperoxidase* (*MPO*) level is correlated with the severity of the disease (47). Others have proposed ATO as a treatment against IBD, showing that it can decrease the expression of *MPO* (48). By associating Cu^2+^ with ATO we have been able to further reduce the expression of *IFN-γ* and *MPO*, thus improving ATO effects. This may suggest that IBD could also benefit from an ATO+Cu^2+^ treatment.

Co-treatment of animals with ATO+Cu^2+^ induced a drop in memory/naive T cells, as already observed by Kavian et al. with high doses of ATO in the same mouse model of chronic GvHD (16). Co-treatment also reduced the activation of B lymphocytes. This result agrees with the results reported by Zhao et al., which showed that ATO reduced the number of B lymphocytes in a mouse model of xenotransplantation (41). The addition of Cu^2+^ to ATO reduced the activation of B lymphocytes, which have an important role in the development of chronic GvHD *via* the production of antibodies but also because of their production of cytokines and chemokines (49). In addition, the co-treatment also inhibited macrophage function by decreasing MHC II^+^ macrophages but also by inducing an M1-type phenotype. M1 and M2 macrophages play a key role in the pathogenesis of cGvHD. It is well known that the activation state of macrophages is important, especially in the context of disorders involving concomitant activation of pro-inflammatory M1 macrophages and profibrotic M2 macrophages (50, 51). Finally, the co-treatment ATO+Cu^2+^ also induced a decrease in the number of activated dendritic cells (CD80^+^), as already observed in other studies, where a high dose of sodium arsenite altered the differentiation and function of dendritic cells *in vitro* (52). Interestingly, we found that our co-treatment limited the over-activation of allogeneic donor immune cells, which is known to play a key role in the development of cGvHD *in vivo*.

Hematopoietic stem cell transplant, which can be the cause of cGvHD, is used to treat many malignant and non-malignant hematological disorders. Therefore, we verified that the anti-fibrotic and immune modulations induced by ATO+Cu^2+^ were not accompanied by an increased potential for tumor growth. ATO, whether alone at high or low dose or in co-treatment with copper, did not influence tumor development of B-cell lymphoma cells injected subcutaneously in all groups of mice.

Our data establish the therapeutic effect of the combination of low dose ATO associated with copper ions, in a mouse model of sclerodermatous cGvHD (graphical abstract). Increasing the production of ROS by adding low concentrations of copper to low doses of ATO contributed to limiting the development of the disease. By combining copper with ATO we were able to treat mice with half of the amount of ATO, thus potentially decreasing or abrogating part of its expected harmful effects. The use of a lower dose of ATO for identical effects could allow patients to be treated for a longer period with lower side effects. In addition, we used a copper dosage equivalent to the one used as a food supplement, thus avoiding most if not all of the negative consequences of excess copper (53).

Finally, one of the limitations of this study is that our co-treatment was only tested in a single mouse model. The potential effect of ATO+Cu^2+^ treatment on another systemic autoimmune fibrotic disease should be tested to confirm our results.

## Data availability statement

The raw data supporting the conclusions of this article will be made available by the authors, without undue reservation.

## Ethics statement

The animal study was reviewed and approved by CEEA34.

## Author contributions

Conceptualization, Methodology, FR and FB; Investigation, CC and MT; Formal analysis, CC, CN, MT, DR-G, and FB; Writing –Original Draft, CC, CN, and MJ; Writing –Review CC, CN, MJ, FR, DR-G, and FB; Funding Acquisition, FR; Supervision, FB and CN. All authors contributed to the article and approved the submitted version.

## Funding

This work was supported by grants from MEDSENIC SAS (N201062A10).

## Acknowledgments

The authors thank Véronique Pomi from MEDSENIC, Strasbourg, France, for encouragement, discussions and additional funding. We would also like to thank the Cytometry and Immunobiology (CYBIO) platform of Cochin Institute, Paris, for flow cytometry, for the determination of biomarkers and data analysis, and the HistIM platform of Cochin Institute, Paris, for staining of sections and scanning/interpreting of slides. We are very grateful to Mr. Nick Barton for the thorough proofreading of this manuscript.

## Conflict of interest

FR and FB are listed inventors for an early patent application family (designation) relative to the synergic use of arsenic salts and metallic ions for the treatment of autoimmune diseases. DR-G and FR are currently employees of MEDSENIC SAS.

The remaining authors declare that the research was conducted in the absence of any commercial or financial relationships that could be construed as a potential conflict of interest.

## Publisher’s note

All claims expressed in this article are solely those of the authors and do not necessarily represent those of their affiliated organizations, or those of the publisher, the editors and the reviewers. Any product that may be evaluated in this article, or claim that may be made by its manufacturer, is not guaranteed or endorsed by the publisher.

## Supplementary material

Supplementary Figure 1
**(A–F)** Standardized H_2_O_2_ level produced by HL-60 cells in culture after treatment during 48 hours with one concentration of ATO (1 µM) and with increasing concentrations of FeSO_4_ (0-4 µM) or AuCl_2_ (0-1 µM) or MnSO_4_/MnCl_2_ (0-1 µM) or ZnSO_4_/ZnCl_2_ (0-50 µM). H_2_DCFDA was measured by spectrofluorometry and the results (AU) were divided par cell viability established by colorimetry (crystal violet). NS: not significant; *p <0.05; **p<0.01; ***p<0.001; ****p<0.0001. The results are the mean of 6-plicates per sample.

Supplementary Figure 2
**(A–F)** Viability of HL-60 cells in culture measured after treatment during 48 hours with one concentration of ATO (1 µM) and with increasing concentrations of FeSO_4_ (0-4 µM) or AuCl_2_ (0-1 µM) or MnSO_4_/MnCl_2_ (0-1 µM) or ZnSO_4_/ZnCl_2_ (0-50 µM). The results are the mean of 6-plicates per sample.

Supplementary Figure 3
**(A)** Standardized H_2_O_2_ level produced by A20 cells in culture after treatment during 48 hours with one concentration of ATO (1 µM) and with increasing concentrations of CuCl_2_ (0-4 µM). H_2_DCFDA was measured by spectrofluorometry and the results (AU) were divided by cell viability established by colorimetry (crystal violet). **(B)** Standardized GSH level produced by A20 cells in culture after treatment during 48 hours with one concentration of ATO (1 µM) and with increasing concentrations of CuCl_2_ (0-4 µM). The results obtained by spectrofluorometry using monochlorobimane were divided by cell viability established by colorimetry (crystal violet). **(C)** Viability of A20 cells in culture measured after treatment during 48 hours with one concentration of ATO (1 µM) and with increasing concentrations of CuCl_2_ (0-4 µM). ***p<0.001. The results are the mean of 6-plicates per sample.

Supplementary Figure 4
**(A)** Hematoxylin and eosin (H&E) staining of liver sections (5 μm) (Eclipse 80i microscope; Nikon, original magnification ×20). **(B)** Relative mRNA expression of *CD45* in the skin. Data are presented as 2^(−ΔΔCT)^ relative to the levels of *GAPDH*. *p <0.05; ***p<0.001; ****p<0.0001. The results for each group are the mean of the measurement obtained per mouse: syngeneic (n = 7); cGvHD (n = 10); cGvHD-Cu (n = 6); cGvHD-ATO 2.5 µg/g (n = 8); cGvHD-ATO-Cu (n = 11); cGvHD-ATO 5 µg/g (n = 5). *Ex vivo* measurements were realized in duplicate for each mouse.

Supplementary Figure 5
**(A)** Flow cytometry gating strategy to detect splenic CD4^+^, CD8^+^ T cells and B cells from BALB/c mice for further analysis of surface marker expression. CD4^+^ T cells were identified as CD3^+^ CD4^+^ double positive cells and CD8^+^ T cells were identified as CD3^+^ CD8^+^ double positive cells. B cells were identified as CD3^-^ B220^+^. **(B)** Flow cytometry gating strategy to identify splenic macrophages and dendritic cells from BALB/c mice for further surface marker analysis. Splenic macrophages were identified as CD11b^+^ and F4/80^+^ double positive cells and dendritic cells were identified as CD11b^+^ and CD11c^+^ double positive cells.

Supplementary Figure 6
**(A)** Tumor volume evolution in the different groups from the first day of injection of the treatments until day 20. Tumor evolution was monitored twice a week with a microcaliper and tumor volumes were calculated as length x (width)^2^ x 0.5 and expressed in mm^3^. **(B)** Tumor volume evolution in the different groups at day 20. The results for each group are the mean of the measurement obtained per mouse: A20-PBS (n = 8); A20-Cu (n = 9); A20-ATO 2.5 µg/g (n = 9); A20-ATO-Cu (n = 9); A20-ATO 5 µg/g (n = 9).

Supplementary Table 1 List of primer sequences used for real-time quantitative polymerase chain reaction.
