# Supplementary figures and images for: A Fenton-like cation can improve arsenic trioxide treatment of sclerodermatous chronic Graft-versus-Host Disease in mice
Source: Front Immunol. 2022 Aug 9;13:917739. doi: 10.3389/fimmu.2022.917739 (PMC9395715; doi:10.3389/fimmu.2022.917739)

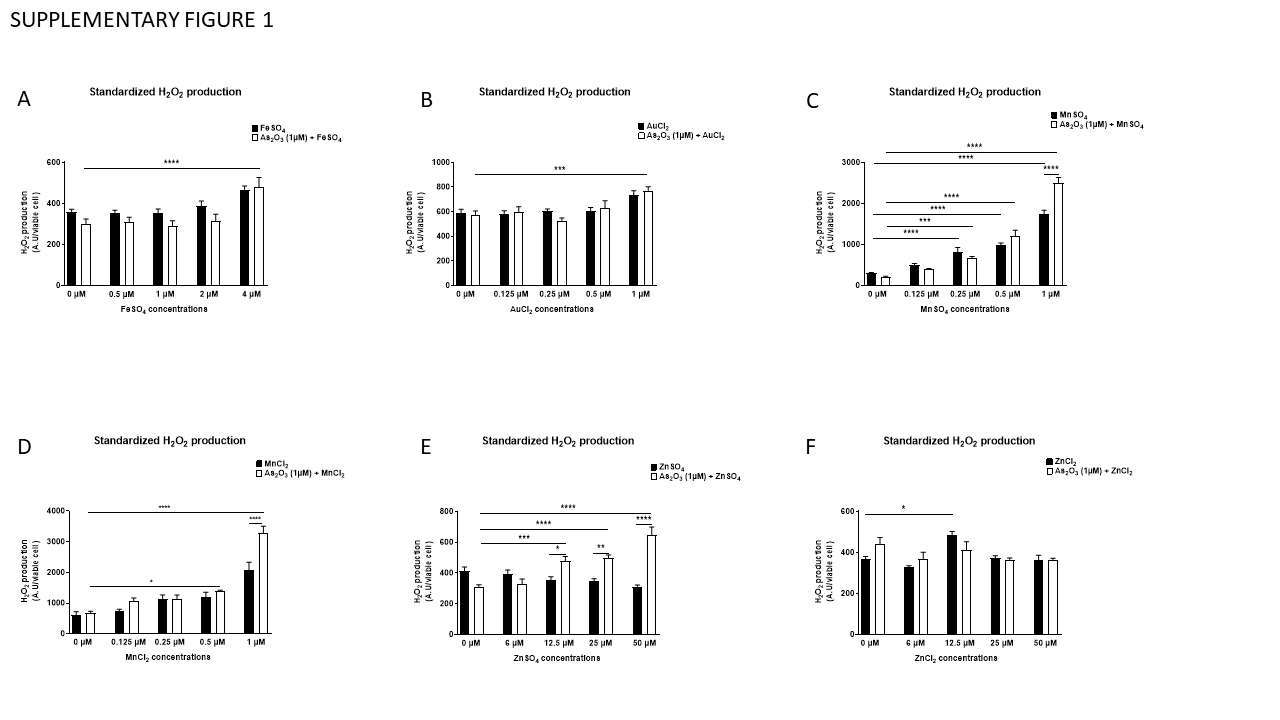

Supplement: Supplementary Figure 1 — (A–F) Standardized H2O2 level produced by HL-60 cells in culture after treatment during 48 hours with one concentration of ATO (1 µM) and with increasing concentrations of FeSO4 (0-4 µM) or AuCl2 (0-1 µM) or MnSO4/MnCl2 (0-1 µM) or ZnSO4/ZnCl2 (0-50 µM). H2DCFDA was measured by spectrofluorometry and the results (AU) were divided par cell viability established by colorimetry (crystal violet). NS: not significant; *p <0.05; **p<0.01; ***p<0.001; ****p<0.0001. The results are the mean of 6-plicates per sample. [file Image_1.tif]

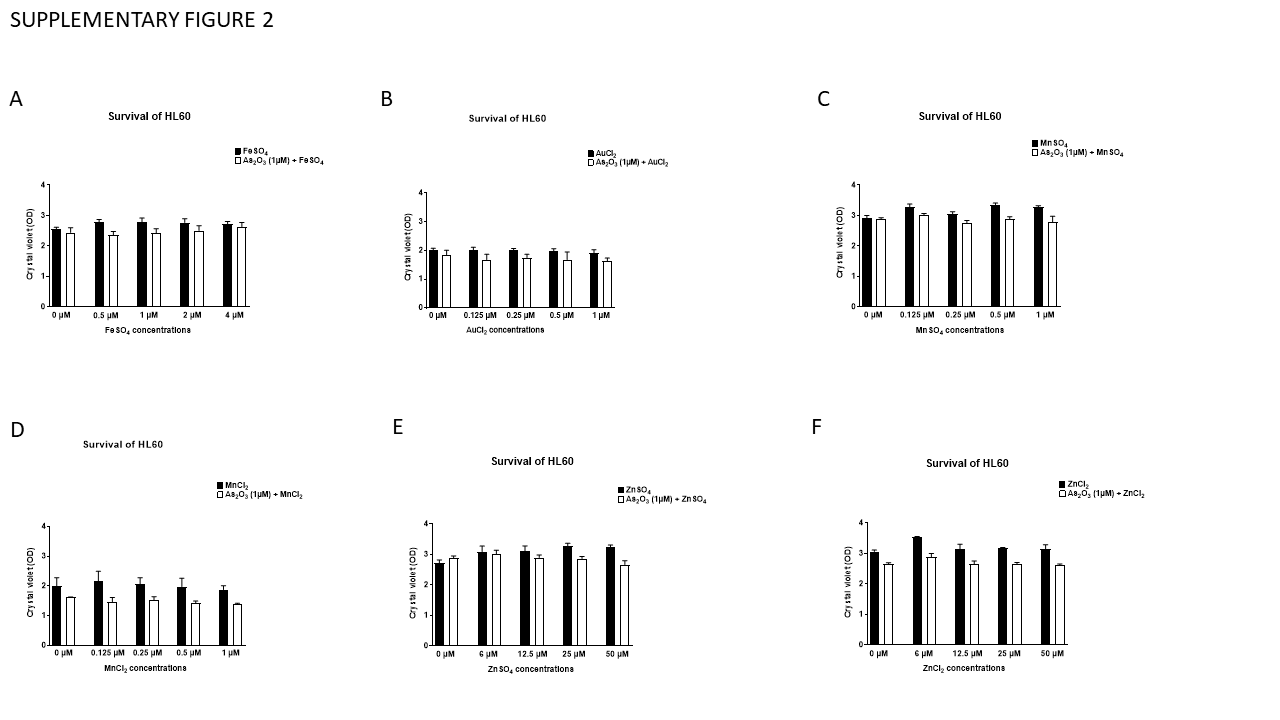

Supplement: Supplementary Figure 2 — (A–F) Viability of HL-60 cells in culture measured after treatment during 48 hours with one concentration of ATO (1 µM) and with increasing concentrations of FeSO4 (0-4 µM) or AuCl2 (0-1 µM) or MnSO4/MnCl2 (0-1 µM) or ZnSO4/ZnCl2 (0-50 µM). The results are the mean of 6-plicates per sample. [file Image_2.tif]

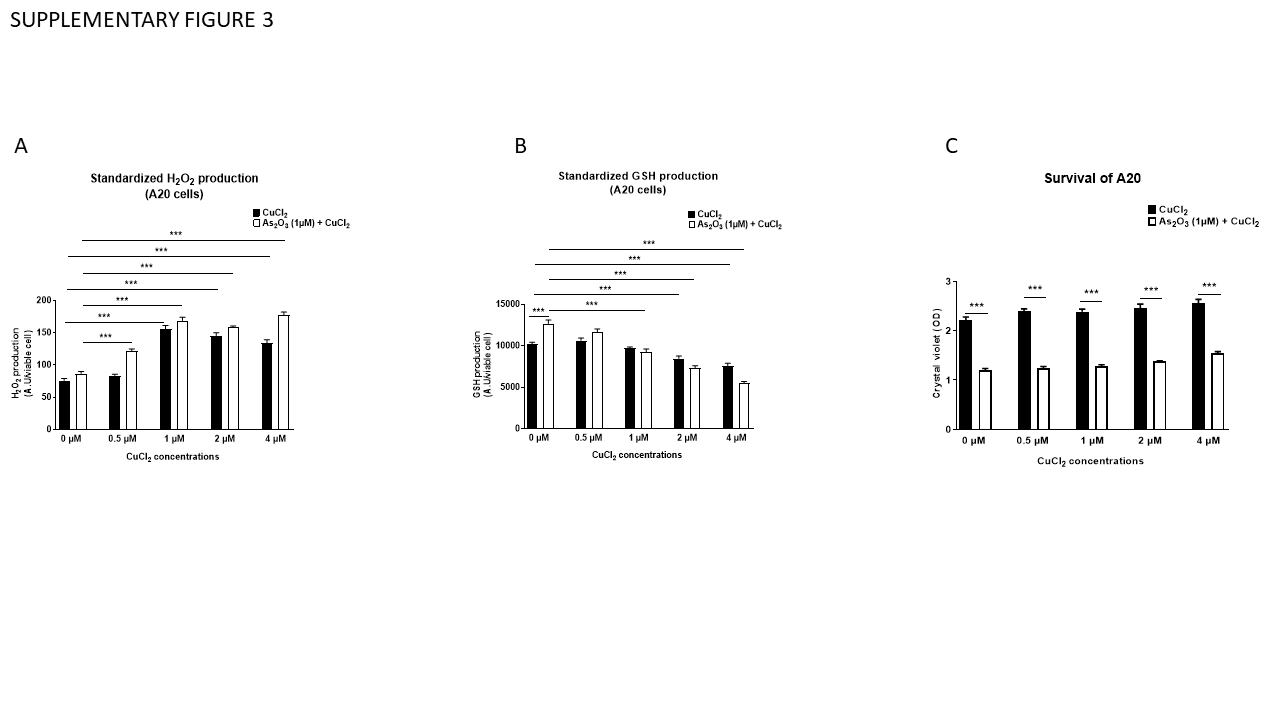

Supplement: Supplementary Figure 3 — (A) Standardized H2O2 level produced by A20 cells in culture after treatment during 48 hours with one concentration of ATO (1 µM) and with increasing concentrations of CuCl2 (0-4 µM). H2DCFDA was measured by spectrofluorometry and the results (AU) were divided by cell viability established by colorimetry (crystal violet). (B) Standardized GSH level produced by A20 cells in culture after treatment during 48 hours with one concentration of ATO (1 µM) and with increasing concentrations of CuCl2 (0-4 µM). The results obtained by spectrofluorometry using monochlorobimane were divided by cell viability established by colorimetry (crystal violet). (C) Viability of A20 cells in culture measured after treatment during 48 hours with one concentration of ATO (1 µM) and with increasing concentrations of CuCl2 (0-4 µM). ***p<0.001. The results are the mean of 6-plicates per sample. [file Image_3.tif]

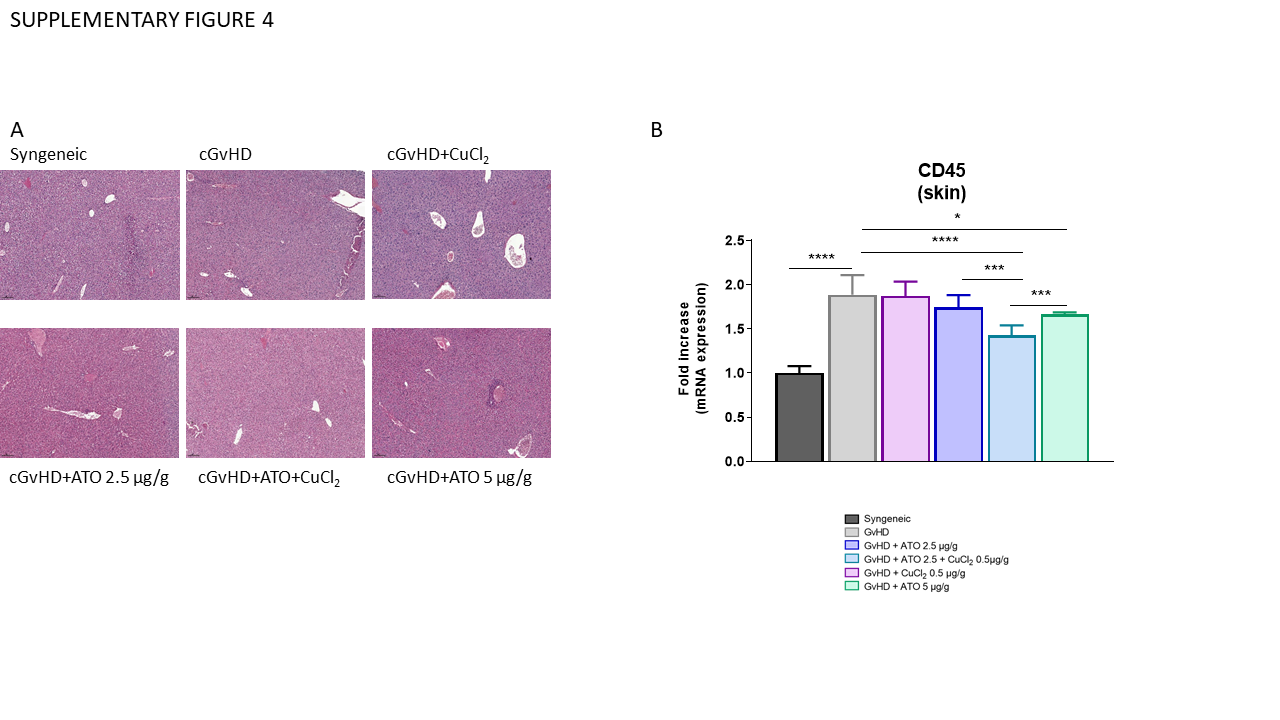

Supplement: Supplementary Figure 4 — (A) Hematoxylin and eosin (H&E) staining of liver sections (5 μm) (Eclipse 80i microscope; Nikon, original magnification ×20). (B) Relative mRNA expression of CD45 in the skin. Data are presented as 2(−ΔΔCT) relative to the levels of GAPDH. *p <0.05; ***p<0.001; ****p<0.0001. The results for each group are the mean of the measurement obtained per mouse: syngeneic (n = 7); cGvHD (n = 10); cGvHD-Cu (n = 6); cGvHD-ATO 2.5 µg/g (n = 8); cGvHD-ATO-Cu (n = 11); cGvHD-ATO 5 µg/g (n = 5). Ex vivo measurements were realized in duplicate for each mouse. [file Image_4.tif]

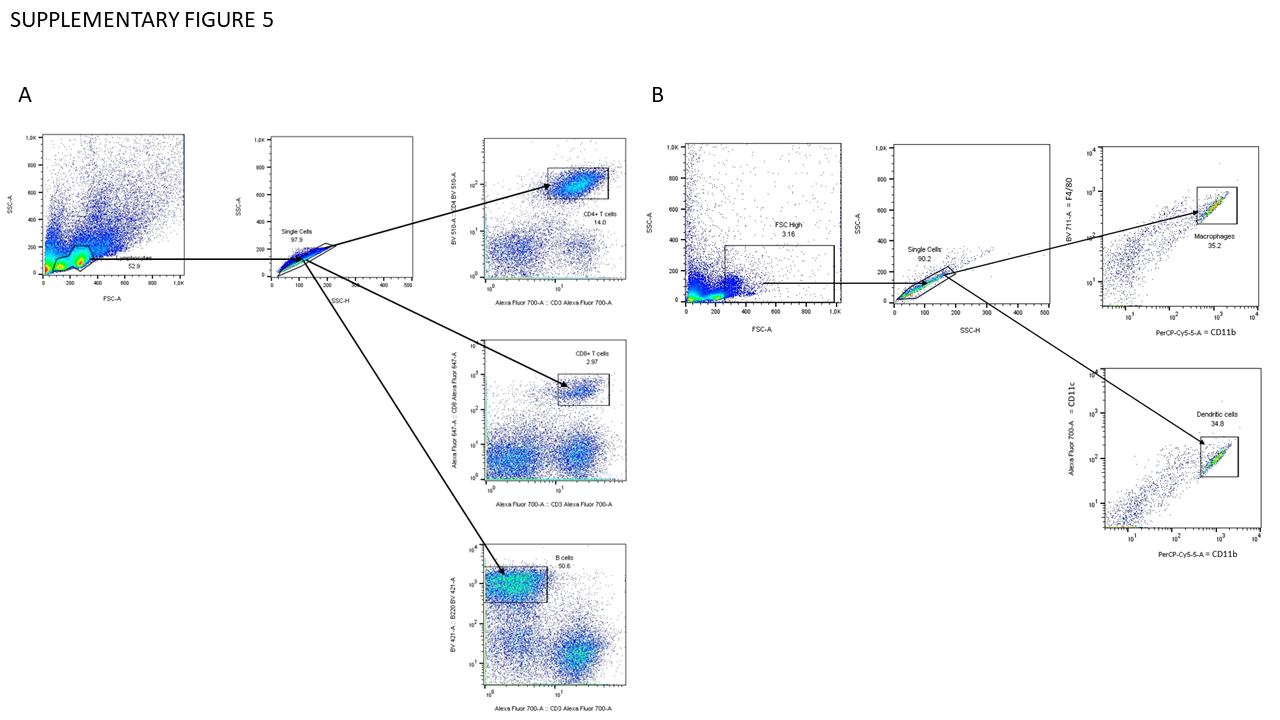

Supplement: Supplementary Figure 5 — (A) Flow cytometry gating strategy to detect splenic CD4+, CD8+ T cells and B cells from BALB/c mice for further analysis of surface marker expression. CD4+ T cells were identified as CD3+ CD4+ double positive cells and CD8+ T cells were identified as CD3+ CD8+ double positive cells. B cells were identified as CD3- B220+. (B) Flow cytometry gating strategy to identify splenic macrophages and dendritic cells from BALB/c mice for further surface marker analysis. Splenic macrophages were identified as CD11b+ and F4/80+ double positive cells and dendritic cells were identified as CD11b+ and CD11c+ double positive cells. [file Image_5.tif]

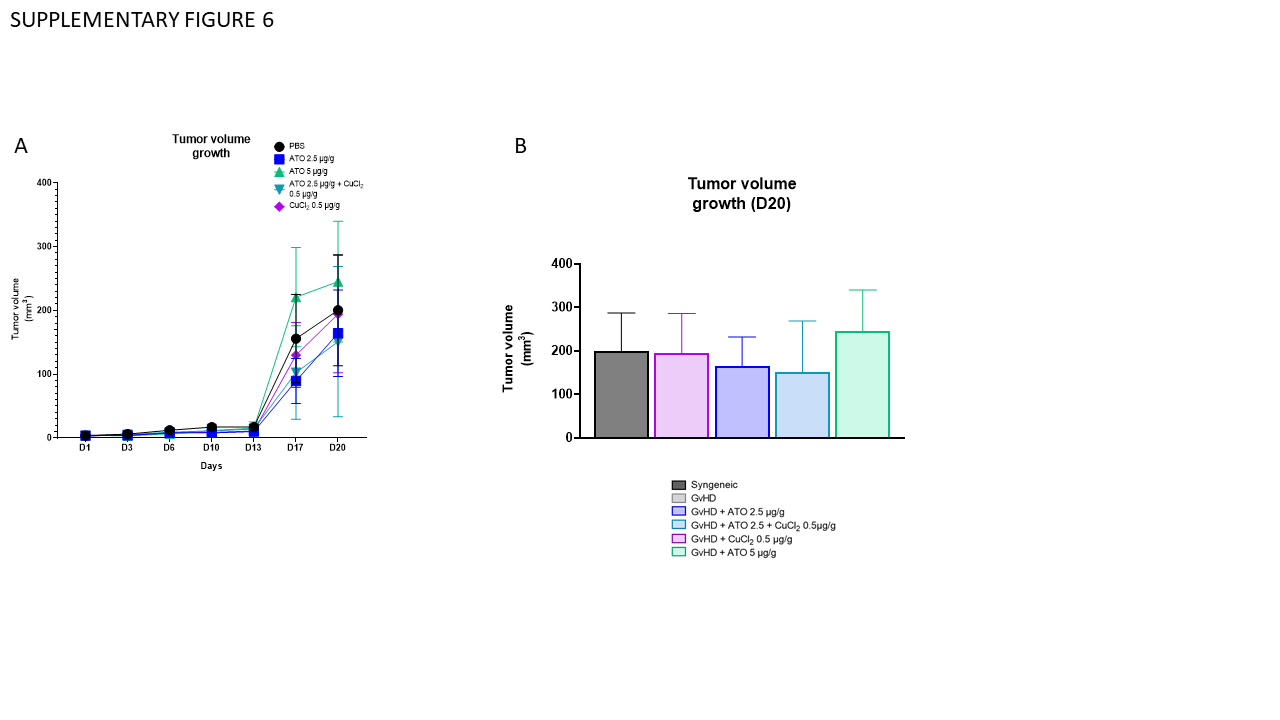

Supplement: Supplementary Figure 6 — (A) Tumor volume evolution in the different groups from the first day of injection of the treatments until day 20. Tumor evolution was monitored twice a week with a microcaliper and tumor volumes were calculated as length x (width)2 x 0.5 and expressed in mm3. (B) Tumor volume evolution in the different groups at day 20. The results for each group are the mean of the measurement obtained per mouse: A20-PBS (n = 8); A20-Cu (n = 9); A20-ATO 2.5 µg/g (n = 9); A20-ATO-Cu (n = 9); A20-ATO 5 µg/g (n = 9). [file Image_6.tif]
