# Supplementary material for: A Fenton-like cation can improve arsenic trioxide treatment of sclerodermatous chronic Graft-versus-Host Disease in mice
Source: Front Immunol. 2022 Aug 9;13:917739. doi: 10.3389/fimmu.2022.917739 (PMC9395715; doi:10.3389/fimmu.2022.917739)
Supplement: Supplementary Table 1 — List of primer sequences used for real-time quantitative polymerase chain reaction. [file Table_1.pdf]

SUPPLEMENTARY TABLE 1

a)

Murine primers used for RT-qPCR		
**Gene promoter**	**Forward sequence**	**Reverse sequence**
**α-SMA**	CTACGAACTGCCTGACGGG	GCTGTTATAGGTGGTTTCGTGG
**β-actin**	ACCACCATGTACCCAGGCATT	CCACACAGAGTACTTGCGCTCA
**Collagen I**	TGTTCGTGGTTCTCAGGGTAG	TTGTCGTAGCAGGGTTCTTTC
**IFN-γ**	ATGAACGCTACACACTGCATC	CCATCCTTTTGCCAGTTCCTC
**IL-13**	CCTGGCTCTTGCTTGCCTT	TGGCGAAACAGTTGCTTTGT
**MPO**	TGAATCCTCGATGGAATGGG	ACGGAAAGCGTTGGTGAAGA
**CD45**	ATGGTCCTCTGAATAAAGCCCA	TCAGCACTATTGGTAGGCTCC
